# Fabrication and modification of implantable optrode arrays for *in vivo* optogenetic applications

**DOI:** 10.1007/s41048-018-0052-4

**Published:** 2018-04-20

**Authors:** Lulu Wang, Kang Huang, Cheng Zhong, Liping Wang, Yi Lu

**Affiliations:** 1 Shenzhen Key Lab of Neuropsychiatric Modulation and Collaborative Innovation Center for Brain Science, CAS Center for Excellence in Brain Science and Intelligence Technology, The Brain Cognition and Brain Disease Institute, Shenzhen Institutes of Advanced Technology, Chinese Academy of Sciences, Shenzhen, 518055 China; 2 Shenzhen College of Advanced Technology, University of Chinese Academy of Sciences, Shenzhen, 518055 China

**Keywords:** Optogenetics, Optrode, Neural electrode, Electrodeposition, Optical stimulation, Electrophysiological recording

## Abstract

**Graphical Abstract:**

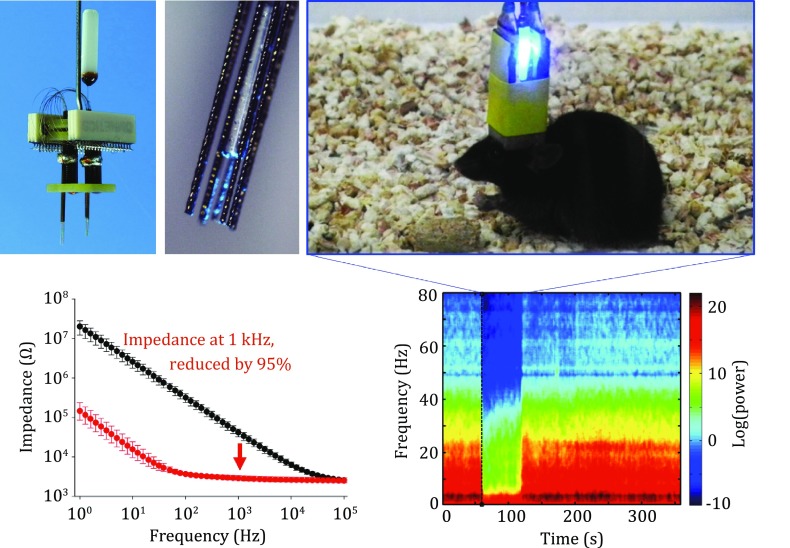

**Abstract:**

Recent advances in optogenetics have established a precisely timed and cell-specific methodology for understanding the functions of brain circuits and the mechanisms underlying neuropsychiatric disorders. However, the fabrication of optrodes, a key functional element in optogenetics, remains a great challenge. Here, we report reliable and efficient fabrication strategies for chronically implantable optrode arrays. To improve the performance of the fabricated optrode arrays, surfaces of the recording sites were modified using optimized electrochemical processes. We have also demonstrated the feasibility of using the fabricated optrode arrays to detect seizures in multiple brain regions and inhibit ictal propagation *in vivo*. Furthermore, the results of the histology study imply that the electrodeposition of composite conducting polymers notably alleviated the inflammatory response and improved neuronal survival at the implant/neural-tissue interface. In summary, we provide reliable and efficient strategies for the fabrication and modification of customized optrode arrays that can fulfill the requirements of *in vivo* optogenetic applications.

## Introduction

Optogenetics is a technology that combines optical control and genetic targeting using cell-type-specific and optically sensitive proteins for the precise manipulation of neuronal functions with millisecond precision (Yizhar *et al*. 2011a; Zhang *et al*. 2007). A major advantage of optogenetic methodology is that it enables the excitation or inhibition of specific neuron subtypes and the electrophysiological recording of neuronal activity simultaneously without causing large stimulus artifacts that may overlap the recording results (Anikeeva *et al*. 2012). Benefiting from these advantages, optogenetics has been widely applied to investigate the mechanisms of neuropsychiatric diseases as well as the functions of brain networks through precisely timed control of specific neuron groups in a neural circuit (Brown 2012; Gradinaru 2009; Kravitz *et al*. 2010; Li 2015; Lu *et al*. 2016; Otis *et al*. 2017; Schmitt *et al*. 2017; Tovote *et al*. 2016; Tye *et al*. 2013; Yizhar *et al*. 2011b; Zimmerman *et al*. 2016).

In order to investigate complex brain processing mechanisms at a functional level, researchers need to use optogenetics to detect the activity from specific neural circuits during the manipulation of target neuron populations. In many cases, chronic studies in freely moving animals are necessary for dissecting the characteristics of a particular neural circuit control animal behavior and understanding the intrinsic neural basis of a specific behavioral phenomenon. Therefore, chronically implantable optrode arrays that integrate optical stimulation with large-scale electrophysiological readout methods are greatly desired for *in vivo* applications (Anikeeva *et al*. 2012; Lu *et al*. 2012). Recent advances in Micro-Electro-Mechanical Systems (MEMS) have shed new light on the development of optrodes (Chen *et al*. 2013; Dehkhoda *et al.* 2018; Iseri and Kuzum 2017; Kwon *et al*. 2015). However, a reliable and efficient fabrication technology for customized chronically implantable optrodes still needs to be developed to meet the requirements of different experimental purposes.

Optrode arrays are typically composed of an optical waveguide for light delivery and multiple electrode channels for recording. The electrode is recognized to be a crucial readout element in optrodes, which determines the signal-recording quality required for neural circuit dissection (Anikeeva *et al*. 2012; Lu *et al*. 2012). However, achieving effective and stable long-term electrophysiological recording, with the electrode *in vivo,* continues to be a great challenge. Therefore, another desirable research goal related to optrodes is to develop novel materials for biocompatible and low-impedance electrode/neural-tissue interfaces.

With the development of microfabrication technologies, high-density microelectrode arrays have been widely used to increase spatial precision and minimize insertion trauma. However, the impedance of the electrode increases drastically with a decrease in its size, which subsequently results in high thermal-background noise and a low signal-to-noise ratio during recording. To solve this problem, electrochemical deposition of high double-layer capacitance or Faradaic-capacitance materials, such as platinum particles, titanium nitride, and iridium oxide, has been frequently used to reduce the electrode impedance (Cogan 2008; Kotov *et al*. 2009; Lu *et al*. 2009b). Another major bottleneck that unfortunately hinders the applications of neural probes is the inconsistent performance caused by elicited inflammatory response (Grill *et al*. 2009; Kotov *et al*. 2009; Schwartz *et al*. 2006). The inflammatory response usually results in a dense astroglial encapsulation of the implant, which isolates the neural probe from the surrounding neurons, and the response also leads to a loss of neurons adjacent to the electrode/neural-tissue interface, which further deteriorates the performance of the electrode in chronic studies. A commonly applied strategy for improving both the electrochemical performance and biocompatibility of neural electrodes is surface modification with conducting polymers (CPs). Previous studies have reported that CPs such as polypyrrole (PPy) and poly(3,4-ethylenedioxythiophene) (PEDOT) can notably increase the electrode capacitance, reduce surface impedance, and alleviate inflammatory responses (Abidian and Martin 2008; George *et al*. 2005; Lu *et al*. 2010; Zhong *et al*. 2017). However, the electrodeposition process and characteristics of these materials have not been adequately investigated with respect to the requirements of optogenetics.

In this work, we report on the fabrication of two types of optrode arrays for *in vivo* optogenetic applications, including a microwire optrode array for investigating single brain regions and a drivable optrode array for simultaneously targeting multiple brain structures. In order to improve the performance of the fabricated optrodes during electrophysiological recording, we also demonstrate three different strategies for decreasing the electrode impedance: a simplified cathodic electrochemical process for the deposition of platinum particles, a cyclic voltammetric process for depositing iridium oxide, and a multi-step modification process for composite CP deposition. The electrochemical characteristics and surface morphology of the deposited optrodes were determined by performing cyclic voltammetry (CV), electrochemical impedance spectroscopy (EIS), and scanning electron microscopy (SEM) analyses. Furthermore, we examined the feasibility of using customized optrode arrays to investigate the neuronal activity during the ictal period and inhibit seizure propagation in multiple brain regions *in vivo*. Finally, after chronic implantation in rat brains, the astrocyte intensity and neuronal survival around the modified implant sites were assessed by analyzing the immunoreactivity of glial fibrillary acidic protein (GFAP) and neuronal nuclei (NeuN), respectively. All of these results were evaluated and discussed with respect to the requirements of optogenetic applications.

## Experimental section

### Animals

All experiments were conducted in accordance with the protocols approved by the Ethics Committee for Animal Research, Shenzhen Institute of Advanced Technology, Chinese Academy of Sciences. Male Sprague–Dawley (SD) rats weighting 240–280 g were used for optrode implantation and histological study. Adult male VGAT-ChR2(H134R)-EYFP bacterial artificial chromosome (BAC) transgenic mice were used for optogenetic stimulation and electrophysiological recordings. Animals were housed in controlled conditions (ambient temperature 24 ± 1 °C, humidity 50%–60%, 12-h light/dark cycle) with food and water given *ad libitum*.

### Fabrication of optogenetic probes

#### Fabrication of microwire optrode arrays

**Fig. 1:**
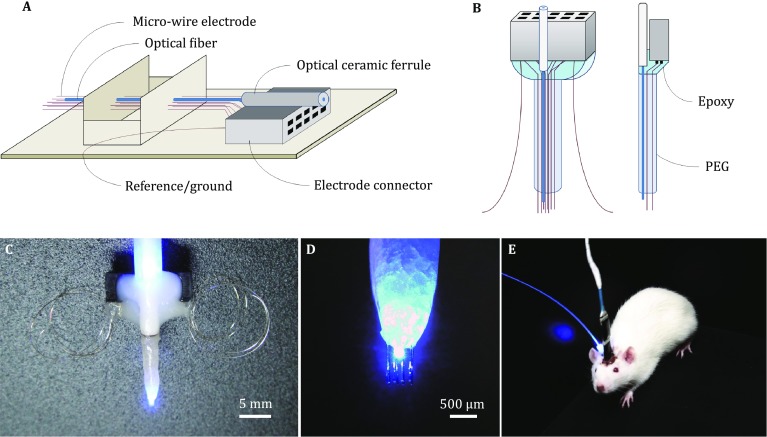
Fabrication of microwire optrode arrays. **A** Fabrication process for an eight-channel microwire optrode array using a custom-designed mold. **B**, **C** A schematic diagram (**B**) and photo (**C**) of an eight-channel microwire optrode array. **D** Tip of the microwire optrode array (enwrapped by PEG). **E** Optogenetic stimulation and electrophysiological recording using a microwire optrode array implanted in a free-moving rat

Microwire optrode arrays were fabricated from optical fibers (diameter 200 μm, NA 0.37, Thorlabs, USA) and formvar-coated nickel–chromium (diameter 35.6 μm, Stablohm 650, California Fine Wire, USA) or platinum–iridium (diameter 35.6 μm, Pt/Ir 90/10, California Fine Wire, USA) microwires using a custom-made optrode mold (Fig. 1A). Eight microwires in an optrode array were arranged in two parallel rows for electrophysiological recordings, with each row containing four wires. The spacing between neighboring microwires was 180 μm. Approximately 2 mm of the formvar coating was removed from one end of each microwire by brief exposure to a flame, and each microwire was soldered into separate slots of a standard electrode connector (Omnetics, USA). Two pairs of uninsulated silver microwires (diameter 100 μm) were soldered into the electrode connector as the reference and ground electrodes, respectively. An optical fiber was arranged to deliver light using the optrode mold. One end of the optical fiber was fixed to an optical ceramic ferrule (inner diameter = 220 µm) and stabilized onto the electrode connector using epoxy resin (Fig. 1B). The tip of the optrode was coated with polyethylene glycol (PEG, *M*_W_ = 2000 g/mol, Sigma–Aldrich, USA) to enhance its mechanical strength (Fig. 1C). Prior to implantation, phosphate-buffered saline (PBS) was applied to dissolve and remove the PEG coatings. In order to ensure illumination of the neurons to be recorded, the tips of the microwires were placed approximately 500 µm deeper than the optical fiber (Fig. 1D). This microwire optrode array is easy to fabricate and is suitable for targeting single regions in relatively shallow brain structures (Fig. 1E).

#### Fabrication of multisite drivable optrode arrays

**Fig. 2:**
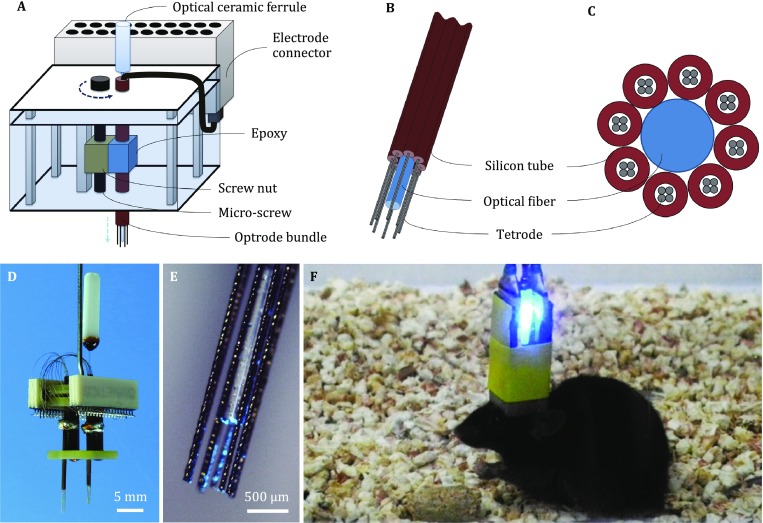
Design of multisite drivable optrode arrays. **A** Schematic diagram of a 32-channel drivable optrode array. **B**, **C** Detailed schematic diagram (**B**) and top view (**C**) of the optrode tip. **D**, **E** Photos of a 64-channel multisite drivable optrode array (**D**) and the optrode tip (**E**). **F** Optogenetic stimulation and electrophysiological recording using a multisite drivable optrode array implanted in a freely moving mouse

The drivable optrode arrays consisted of screw-based microdrive scaffolds (Fig. 2A) and optrode bundles (Fig. 2B). Each optrode bundle, which contains one stimulation channel and eight twisted tetrodes (32 electrophysiological recording channels in total), was fabricated from optical fibers (diameter 200 μm, NA 0.37, Thorlabs, USA) and formvar-coated nickel–chromium (diameter 12.7 or 17.8 μm, Stablohm 650, California Fine Wire, USA) or platinum–iridium (diameter 12.7 or 17.8 μm, Pt/Ir 80/20, California Fine Wire, USA) microwires. Each tetrode was threaded through a silica tube (inner diameter 100 µm, outer diameter 164 µm, Polymicro, USA). In an optrode bundle, eight tetrodes were arranged around an optical fiber, and the center-to-center spacing between each tetrode and the optical fiber was approximately 200 μm (Fig. 2C). Then, the optrode bundles were glued onto the movable screw nut on the microdrive scaffolds. Additional optrode bundles can be integrated with the microdrive system to construct a multisite drivable optrode array (Fig. 2D–F).

### Electrodeposition

#### Electrodeposition of Pt

All electrochemical experiments were performed using a potentiostat (Gamry Reference 600, USA). The electrodes were mounted in a three-electrode cell with a saturated calomel electrode (SCE) as the reference electrode and a large-area platinum electrode as the counter electrode. The electrodeposition solution was prepared by dissolving PtCl_4_ in 100 mmol/L HCl solution to a concentration of 5 mmol/L. The prepared solution can be used for at least six months after preservation at 4 °C. Platinum nanoparticles were deposited on the recording sites of the optrode in the deposition solution via a simplified cathodic reaction at a potential of −0.25 V (vs. SCE) applied by a Gamry potentiostat. The electrodeposition process can be described by the following equation:

1 $${\text{Pt}}^{4 + } + 4{\text{e}}^{ - } = {\text{ Pt}}.$$

#### Electrodeposition of iridium oxide (IrO_2_)

**Fig. 3:**
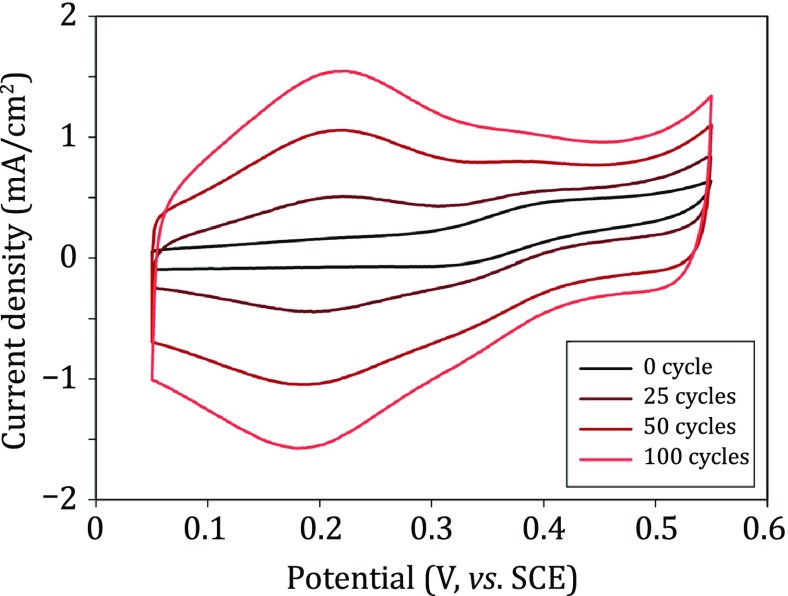
The deposition of IrO_2_ under repetitive potential cycling between 0.05 and 0.55 V (vs. SCE) in an iridium complex ([Ir(OH)_4_C_2_O_4_]^2−^) solution

The electrodeposition solution was prepared as described in previous studies (Lu *et al*. 2009b; Zhong *et al*. 2014). Briefly, oxalic acid (H_2_C_2_O_4_) was slowly dissolved to a concentration of 100 mmol/L in a 3 mmol/L hydrogen hexachloroiridate (H_2_IrCl_6_) aqueous solution, and H_2_O_2_ (2% *v*/*v*) was added to form a stable complex of iridium oxide. Then, the pH of the solution was adjusted to 10.5 by gradually adding K_2_CO_3_. The resulting solution was allowed to age for one week at room temperature until it turned dark blue. The deposition solution can be used for more than two months after preservation at 4 °C. Thin IrO_2_ films were formed on the recording sites in the stabilized electrodepositing solution by cyclic voltammetry scanning between 0.05 and 0.55 V (vs. SCE) at 100 mV/s with a Gamry potentiostat (Fig. 3). The electrodeposition process can be described by the following equation (Lu *et al*. 2009b):

2 $$\left[ {{\text{Ir}}\left( {\text{OH}} \right)_{ 4} {\text{C}}_{ 2} {\text{O}}_{ 4} } \right]^{ 2- } - 2 {\text{e}}^{ - } = {\text{ IrO}}_{ 2} + 2 {\text{CO}}_{ 2} + 2 {\text{H}}_{ 2} {\text{O}}.$$

#### Electrodeposition of composite conducting polymers (CPs)

Poly(vinyl alcohol)/poly(acrylic acid) interpenetrating polymer networks (PVA/PAA IPNs) were synthesized following the approach described in previous studies (Lu *et al*. 2012; Zhong *et al*. 2017). Briefly, an aqueous PVA solution (2 wt%, *M*_W_ 89,000–98,000 g/mol, Sigma–Aldrich, USA) was placed in a round-bottom flask, and acrylic acid (AA) monomer was added under magnetic stirring to a ratio of 60 mol% (moles of AA monomer per mole of PVA repeat unit). Ammonium persulfate (Degussa-AJ, China) was added to the flask to a concentration of 1000 ppm as an initiator. Then, the mixed solution was purged with Ar to remove O_2_, and the flask was sealed and immersed in an oil bath at 80 °C. The reaction was allowed to take place for 72 h, and the resulting polymer solution was filtered to remove undissolved solids and bubbles prior to use. The samples were dipped into the homogeneous polymer solution and dried at 60 °C for 2 h to form a thin hydrogel film on the surface. The aqueous electrodeposition solution was prepared by dissolving 10 mmol/L 3,4-ethylenedioxythiophene monomer (EDOT, Sigma–Aldrich, USA), 250 mmol/L poly(sodium 4-styrenesulfonate) (PSSNa, *M*_W_ 70,000 g/mol, Sigma–Aldrich, USA), 10 μg/mL nerve growth factor (NGF, Sigma–Aldrich, USA), and 10 mmol/L dexamethasone sodium phosphate (DEX, NIFDC, China). The coated substrates were then immersed in the electrodeposition solution for at least 1 h prior to use. PEDOT/PSS/NGF/DEX films were electrodeposited onto the hydrogel-coated substrates using a Gamry potentiostat at 0.9 V (vs. SCE), forming the composite CP films.

### Physicochemical characterizations

Cyclic voltammetry (CV) measurements were performed within the safe potential window in a testing solution at a scan rate of 50 mV/s using a Gamry potentiostat. Electrochemical impedance spectra (EIS) of the recording sites were measured at their open circuit potentials in artificial cerebrospinal fluid (ACSF) using a Gamry potentiostat with a 25-mV (rms) AC sinusoid signal in the frequency range 100 kHz to 1 Hz. The morphologies of the electrodeposited surfaces were examined using an extremely high-resolution scanning electron microscope (XHRSEM, FEI Magellan 400L, USA) operated at 5 kV. In order to facilitate the observation of the modified surfaces, the recording sites of the optrodes were coated with epoxy. The samples were sputtered with a layer of iridium prior to use.

#### Characterization of Pt-deposited electrodes

**Fig. 4:**
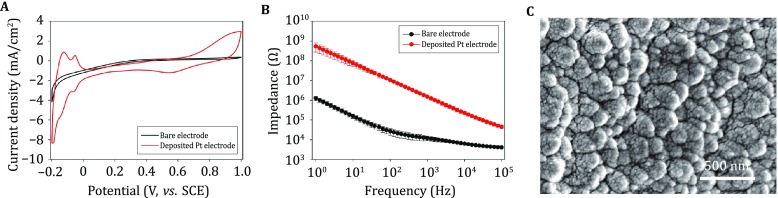
Characterization of platinum particles. **A** Cyclic voltammogram of an optrode before (*black*) and after platinum deposition (*red*) at a sweep rate of 50 mV/s in 0.1 mol/L HCl. **B** Bode plot of electrochemical impedance spectra of an optrode before (*black*, *n* = 4) and after platinum deposition (*red*, *n* = 4) in ACSF, data are shown as mean ± SD. **C** SEM image of the optrode tip after platinum deposition

After Pt electrodeposition, the redox currents in the CV curves were significantly increased (Fig. 4A), suggesting an enlarged double-layer capacitance. The electrochemical impedance at 1 kHz is a critical characteristic parameter for neural electrodes, as this frequency is relevant to the electrical activity of neurons. The average impedance value at 1 kHz was decreased from 1.52 MΩ to 11.96 kΩ, ~99% lower than the unmodified electrode (Fig. 4B), possibly due to the increase in pseudo-capacitance after modification. The surface of the Pt-deposited layer contains numerous nanoparticles that aggregated into sub-micrometer structures. This rough surface significantly increased the effective surface area at the electrode/electrolyte interface and, as a consequence, decreased the electrochemical impedance of the electrode (Fig. 4C).

#### Characterization of IrO_2_-deposited electrodes

**Fig. 5:**
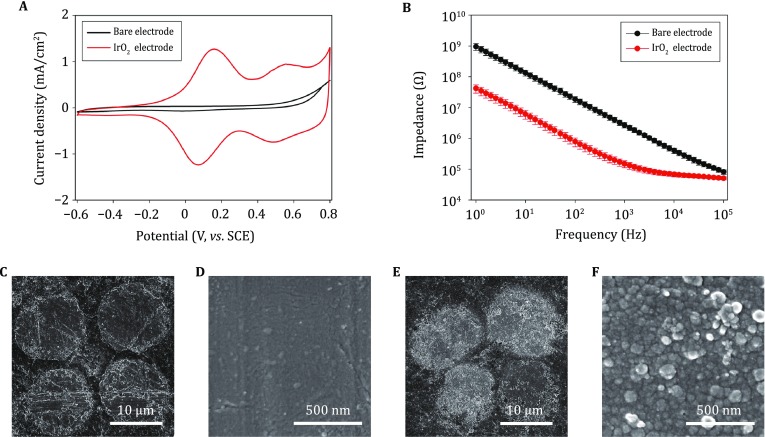
Characterization of IrO_2_. **A** Cyclic voltammogram of an optrode before (*black*) and after IrO_2_ deposition (*red*) at a sweep rate of 50 mV/s in PBS. **B** Bode plot of electrochemical impedance spectra of an optrode before (*black*, *n* = 4) and after IrO_2_ deposition (*red*, *n *= 4) in ACSF, data are shown as mean ± SD. **C**–**F** SEM images of the optrode tip before (**C**, **D**) and after IrO_2_ deposition (**E**, **F**)

No significant current peak was observed in the background CV curve of the undeposited electrode. After cyclic voltammetric scanning in the electrodeposition solution for 100 cycles, two pairs of peaks correlating to redox reactions of iridium oxides appeared at 100 and 500 mV, respectively (Fig. 5A). Besides, the electrode impedance at 1 kHz decreased from 2.71 MΩ to 148 kΩ, ~95% lower than that of the unmodified electrode (Fig. 5B). The surface of the electrode substrate presented a homogeneous and flat morphology (Fig. 5C, D). By comparison, the deposited IrO_2_ film was composed of numerous 20–50 nm particles, and the film exhibited a more porous structure and increased roughness (Fig. 5E, F), which is beneficial for the improvement of the electrochemical performance.

#### Characterization of composite CP-deposited electrodes

The CV measurement of the composite CP-modified electrode shows the emergence of an anodic peak and a cathodic peak at −300 and −400 mV (vs. SCE), respectively (Fig. 5A). In the high-frequency range (10^2^–10^5^ Hz), the impedance of the composite CP-modified electrode was almost independent of frequency. The average impedance value at 1 kHz was ~95% lower than the impedance of the unmodified electrode (Fig. 5B). The drastic increase in charge-storage capacity and reduction in impedance after modification is possibly due to the increase of the pseudo-capacitance and effective surface area (Fig. 5C) at the electrode/electrolyte interface.

### Optogenetic study

#### Seizure detection and inhibition

VGAT-ChR2(H134R)-EYFP BAC transgenic mice were anesthetized with urethane (1.5 g/kg) and fixed in a standard stereotaxic frame. Holes were drilled through the skull, and the multisite optrodes were implanted. Optrode bundles were directed toward the dentate gyrus/hilus (DGH, stereotaxis atlas coordinates: AP −2.06 mm, ML +1.35 mm, DV +2.10 mm) and primary motor cortex (M1, AP +1.80 mm, ML +1.80 mm, DV +1.80 mm), respectively.

Kainic acid (KA) was dissolved in PBS at 0.30 mg/mL, and then 550 nl of the KA solution was unilaterally injected into the dorsal hippocampus using a micropump to induce an acute status epilepticus. Electrophysiological recordings were performed using a 64-channel neural acquisition processor (Plexon, USA). After the onset of seizures, 473 nm blue light pulses (10 mW/mm^2^, 5 ms pulses at 130 Hz, 1 min on and 5 min off) were directed into the implanted optrode array for optogenetic stimulation.

#### Data analysis

Neural electrophysiological data for all recording channels were bandpass filtered. Multi-unit recordings were high-pass filtered (300 Hz) with a Bessel filter for the detection of spikes. The threshold for spike detection was set to −4.5 SD (standard deviations) and the spike waveforms were measured in a time window 1400 μs long and beginning 150 μs before threshold crossing. Local field potentials (LFPs) were sampled at 1 kHz and bandpass filtered at 1–300 Hz. Data analyses were performed with a custom software written in MATLAB (MathWorks, USA) and NeuroExplorer (Nex Technologies, USA), and the mean-squared values of the multi-unit activities, 60 s before and 300 s after optogenetic stimulation (10 s bins), were calculated. LFPs were filtered in beta (12–30 Hz) and gamma bands (30–80 Hz). The squared values of the filtered signals, 60 s before and 300 s after stimulation (10 s bins), were calculated for each band. The power measured before stimulation was averaged (6 bins, 60 s). The power in each bin measured after stimulation was compared with the average power before stimulation by a one-side paired *t* test to investigate whether optical stimulation reduced LFP power.

### Histological study

#### Sample preparation and implantation procedures

After being anesthetized with 1% phenobarbital sodium solution (1 ml/100 g), the rats were immobilized in a stereotaxic frame for sample implantation. A midsagittal incision was made in the scalp, and two holes were carefully bored in each animal at locations −4.0 mm anterior and ±3.0 mm lateral to the bregma. The brains were slowly implanted with the samples: Pt/Ir dummy probes (diameter 100 μm, uninsulated) as the control group and the composite CP-deposited Pt/Ir dummy probes as the testing group. Finally, the implants were fixed to the skull by gluing the cap to the skull surface with a medical adhesive (Fuaile, China), and the skin was sutured shut with monofilament nylon.

#### Tissue preparation and histological analysis

Six weeks after implantation, the rats were sacrificed and immunohistological analyses were performed as described previously (Lu *et al*. 2009a, 2010; Zhong *et al*. 2017). Briefly, the rats were perfused transcardially with 0.1 mol/L PBS followed by 4% paraformaldehyde in 0.1 mol/L PBS, and the brains were removed and fixed at 4 °C for two days. Subsequently, the block tissue around the implant was paraffin-embedded, and horizontal sections (30 μm thick) were prepared from all the brain samples. Antibodies against GFAP and NeuN (both from Abcam, USA) were used to label astrocytes and mature neurons, respectively. Fluorescence images were obtained using an Olympus IX71 inverted fluorescence microscope. Quantitative analysis was performed using custom software developed in MATLAB. The staining intensities of GFAP and NeuN were calculated as a function of distance up to the implant surface. The results shown are the average intensity profiles of the analyzed area within a distance of 250 µm from the implant/neural-tissue interface. All data are presented as mean ± standard error of the mean. The differences in staining intensities of various implants at the same distance were analyzed by performing independent sample *t* tests using SPSS 16.0 (SPSS, USA).

## Results and discussion

### Fabrication and surface modification of optrode arrays

In order to integrate optogenetic stimulation with electrophysiological readout methods, chronically implantable optrode arrays were designed and fabricated. An optrode mold was custom made for arranging the microwires and optical fibers (Fig. 1). Aided by this mold, the recording and stimulation channels can be customized to different experimental designs, which greatly simplified the fabrication process. In order to record neural activity from a large number of individual cells in relatively deep brain areas, drivable optrode arrays were designed based on previous work (Lin *et al*. 2006). The position of optrode bundles on the microdrive scaffold can be conveniently formatted according to different experimental needs (Fig. 2). This lightweight (typically <2 g) optrode array can be used to explore the causal, temporally precise, and behaviorally relevant interactions of neurons in multiple brain regions of freely moving mice.

As the electrode (recording site) is a key readout element of an optrode, its performance is crucial for optogenetic investigations. For improved spatial resolution and signal quality during electrophysiological recording, neural electrodes must meet the requirements of small size and low interface impedance (Cogan 2008). However, because the electrochemical impedance at the interface is inversely proportional to the square of the electrode diameter, balancing the impedance and the size of a neural electrode is a difficult task.

**Fig. 6:**
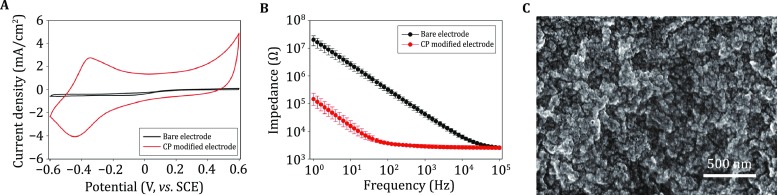
Characterization of conducting polymer (CP) films. **A** Cyclic voltammogram of an optrode before (*black*) and after CP deposition (*red*) at a sweep rate of 50 mV/s in PBS. **B** Bode plot of electrochemical impedance spectra of an optrode before (*black*, *n* = 6) and after CP deposition (*red*, *n* = 6) in ACSF. **C** SEM image of the optrode tip after CP deposition

One of the most commonly applied strategies involves using high-capacitance electroactive materials to decrease the charge-transfer resistance at the electrode/electrolyte (electrode/neural-tissue) interface. Therefore, we modified the recording sites of optrodes with Pt nanoparticles, IrO_2_, and composite CPs, respectively (Figs. 4, 5, 6). After electrodeposition, the redox currents in the cyclic voltammograms of the recording sites were significantly increased, while the electrochemical impedances at 1 kHz were 95%–99% lower than the unmodified sites. This is possibly due to the increase in the pseudo-capacitance after modification, which may be beneficial for decreasing background thermal noise and improving signal quality during electrical recording.

It should be mentioned that the impedance at 100 kHz (*Z*_100kHz_) for the Pt-deposited electrode was approximately 90% lower than that for the undeposited electrode. As the electrode impedance at particularly high frequencies mainly depends on the geometric area of the conductive site, the reduction of *Z*_100kHz_ is probably due to the expanded electrode surface covered with Pt particles after electrodeposition. The expansion of the deposited Pt layers may lead to a short circuit of the neighboring recording sites (especially on tetrodes or other high-density electrode arrays). However, the impedance of the IrO_2_ electrode and the CP electrode at 100 kHz were comparable with that of the undeposited electrode, implying that the IrO_2_ and CP films were not over-expanded during electrodeposition. The electrodepositions of the IrO_2_ and CP films are much more controllable than Pt deposition, which is especially suitable for high-density microelectrode arrays.

### *In vivo* seizure detection and inhibition

**Fig. 7:**
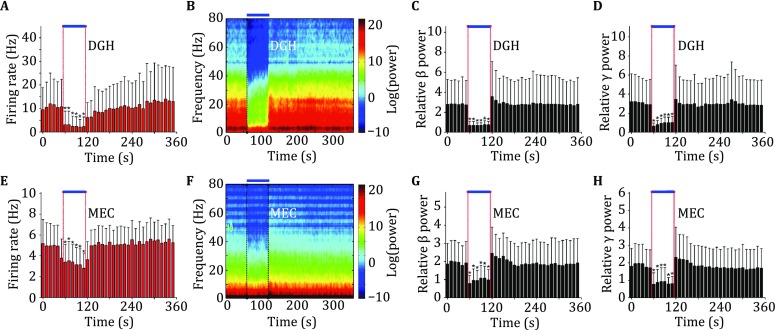
Optogenetic inhibition of seizures in the intrahippocampal kainate-injected mice using a custom-made multisite optrode array. **A**, **E** Averaged multi-unit firing rates recorded in the DG (**A**, *n* = 27 from 4 mice) and M1 (**E**, *n* = 22 from 4 mice). **B**, **F** Representative examples of the spectrograms of LFPs in the DG (**B**) and M1 (**F**). **C**, **D**, **G**, **H** Power quantification of DG (**C**, **D**, *n* = 4 mice) and M1 (**G**, **H**, *n* = 4 mice) LFPs, the averaged powers were shown in the beta (**C**, **G**) and gamma bands (**D**, **H**), and the values were normalized to the total power in the pre-KA period. Light pulses (473 nm, 5-ms pulse duration at 130 Hz) were delivered into the DG/hilus at time 0. The *thick blue line* denotes the 60-s stimulation period. All data are shown as mean ± SEM (*p < 0.05, *t* test)

We next verified the feasibility of using the fabricated optrode arrays to investigate the circuit-level mechanisms controlling seizure activities under the modulation of specific neuron subtypes at high temporal resolution *in vivo*. A customized two-site optrode array was implanted simultaneously into the DGH and M1 of the VGAT-ChR2(H134R)-EYFP transgenic mice. Neural activity in the M1 was recorded as an indicator of behavioral seizures. After the onset of ictal seizures, spontaneous multi-unit bursts and large-amplitude spikes and LFPs were frequently detected in the DGH and M1 (Fig. 7), suggesting hypersynchronization of the affected neurons. We found that selectively activating DGH GABAergic interneurons not only significantly decreased the activity of local neurons (*n* = 27 from 4 mice, Fig. 7A), but also inhibited multi-unit firings in the M1 (*n* = 22 from 4 mice, Fig. 7E). During ictal seizures, the LFP power in the DGH and M1 all remarkably increased within a broad frequency range. Optical stimulation of the DGH GABAergic interneurons caused a significant decrease in local LFP activity, especially at the beta–gamma band (*n* = 4 mice, Fig. 7B–D), as well as in the M1 (*n* = 4 mice, Fig. 7F–H). The neuronal activity levels reduced to control baselines (relative LFP powers are close to 1) during optogenetic modulation, which indicates that activating the DGH inhibitory neurons is sufficient to rescue ongoing behavioral seizures.

### Histological analysis

After a neural electrode is implanted into the central nervous system, a host response is subsequently elicited, which results in the encapsulation of the implant by a dense astrocyte layer that separates the implant from the targeted neurons and hinders charge transfer at the electrode/neural-tissue interface. Consequently, the electrode impedance is increased substantially, which causes a decline in signal quality. Furthermore, the inflammatory response might also lead to a loss of neurons adjacent to the implant/neural-tissue interface, which would further deteriorate the performance of the neural electrodes. Therefore, we used Pt/Ir implants as electrodeposition substrates (the control group); these implants feature large electrode areas and can be used conveniently for investigating the responses of the electrode interface to the host tissue. Previous studies demonstrated that the iridium oxide modified implants exhibited a more significant reactive response compared to platinum substrates *in vivo* (Ereifej *et al*. 2013). Therefore, to improve biocompatibility at the implant/neural-tissue interface, we modified the implants using composite CP films, and then tested each of the deposited and control implants. We performed immunochemical analysis on tissue sections of rat brain at six weeks after implantation and evaluated the chronic performance of the composite CP films.

**Fig. 8:**
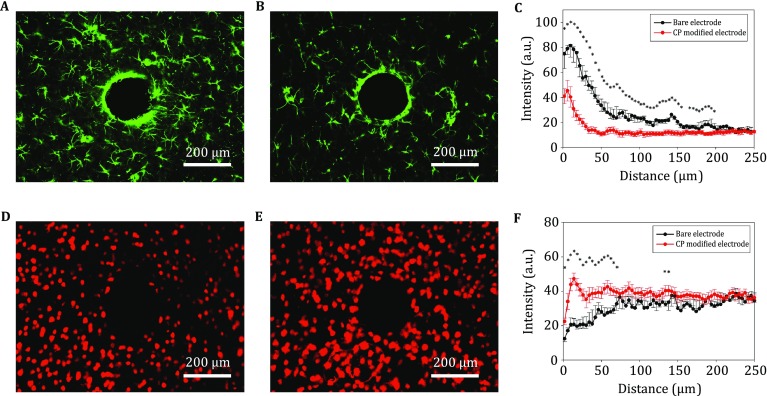
Histological studies of the composite conducting polymer (CP) films’ modified surfaces. Inflammatory response (**A**–**C**, GFAP) and neuronal survival (**D**–**F**, NeuN) around implants at six weeks post implantation in the rat brain before (**A**, **D**) and after composite CP deposition (**B**, **E**). Quantitative comparisons of the immunoreactivity between control (*n* = 7) and CP-modified implants (*n* = 7) were performed by using intensity profiles as a function of distance from the implant interface, shown as mean ± SEM (*p < 0.05, *t* test)

The inflammatory response at the implant/neural-tissue interface was characterized by the expression of GFAP, an astrocyte-specific marker. We found that reactivated astrocytes accumulated in and occupied the zone around the implantation site in the control group (Fig. 8A). By comparison, GFAP expression was considerably weaker around the implantation site in the composite CP group (Fig. 8B). Figure 8C shows the quantified GFAP immunohistological intensity profiles of the control and composite CP-modified implants as a function of distance from the interface (*n* = 7 in each group). The average thickness of the astroglial encapsulation in the control group was ~150 μm, whereas it was only ~40 μm in the CP-modified group. Statistical results show that the average intensity of GFAP immunostaining in the CP-modified group was significantly lower (*p* < 0.05) than that in the control group up to a distance of ~200 µm from the implant interface. Besides, neuronal loss was observed around the implantation site, and this loss was particularly severe in the control group (Fig. 8D). Interestingly, no notable loss of neurons was detected in the composite CP group (Fig. 8E). The quantified NeuN intensity profiles (Fig. 5F, *n* = 7 in each group) reveal a severe loss of neurons within an average distance of ~60 µm from the implantation site in the control group. However, in the CP-modified groups, the average distance was decreased to ~15 µm. Statistical analysis suggests that the average intensity of NeuN immunostaining in the modified group was markedly higher than that in the control group (*p* < 0.05 within ~60 µm). Given the inherent merits of the deposited CP film, our data suggest that modification of the electrode with the composite CP film can drastically alleviate the inflammatory response (reduced astroglial intensity) and promote neuronal viability (increased NeuN intensity) around the implant site. This implies that the composite CP film can improve the implant/neural-tissue interface and is suitable for long-term implantation.

## Conclusion

In this study, we have demonstrated the feasibility and advantages of using customized optrode arrays for *in vivo* optogenetic applications. We designed and fabricated a microwire optrode array and a drivable optrode array, which are suitable for targeting single sites in relatively shallow brain structures and multiple sites in relatively deep structures, respectively. Besides, electrochemical deposition strategies for improving the performance of optrode arrays were also presented, and the physicochemical characteristics of the deposited surfaces were investigated. Platinum particles were deposited by a simplified cathodic electrochemical process, which rapidly decreased the electrode impedance (reduced by 99% at 1 kHz) by increasing the double-layer capacitance at the electrode interface. Low impedance and stable IrO_2_ films were formed on the recording sites under a slow-sweep-rate cyclic voltammetric scanning process. The electrodeposition of IrO_2_ films is highly controllable, which is suitable for high-density optrode arrays. Furthermore, our data suggest that the electrodeposited composite CP films can significantly improve both the electrochemical performance and the biocompatibility of implants. With the aid of our customized optrode arrays, we successfully analyzed the neuronal activity during seizures and inhibited ictal propagation in multiple brain regions *in vivo*. All of these characteristics demonstrated and discussed here are crucial for the chronic optrode arrays used for precisely timed analyses of neuron subpopulations in freely moving animals. Most importantly, our results provide simple and reliable strategies for the fabrication and surface modification of optrode arrays. Combinations of these aforementioned strategies could help to construct high-performance optrode arrays optimized to the requirements of different experimental conditions. Other new fabrication strategies and novel interface materials, as well as future applications of the optrode arrays, are avenues for further investigations.
